# Factors Associated With Intention and Use of e–Mental Health by Mental Health Counselors in General Practices: Web-Based Survey

**DOI:** 10.2196/34754

**Published:** 2022-12-20

**Authors:** Ann E M De Veirman, Viviane Thewissen, Matthijs G Spruijt, Catherine A W Bolman

**Affiliations:** 1 Faculty of Psychology Open University of the Netherlands Heerlen Netherlands; 2 Therapieland Amsterdam Netherlands

**Keywords:** mental health counselors, general practices, e–mental health, adoption readiness, eligibility for e–mental health, e–mental health use, mental health, eHealth

## Abstract

**Background:**

Mental health care counselors have a high intention to use e–mental health (EMH), whereas actual use is limited. Facilitating future use requires insight into underlying factors as well as eligibility criteria that mental health care counselors use in their decision to apply EMH.

**Objective:**

The aim of this study was to unfold the intention and underlying reasons for mental health counselors to use EMH and to unveil the criteria they use to estimate patient eligibility for EMH. The theoretical framework was based on the reasoned action approach model, the Unified Theory of Acceptance and Use of Technology, and the Measurement Instrument for Determinants of Innovation model.

**Methods:**

To empirically validate our theoretical model, a web-based survey was conducted among mental health care counselors (n=132). To unveil the eligibility criteria, participants were asked to rank their reasons for considering EMH suitable or unsuitable for a patient.

**Results:**

The mean intention to use EMH was positive (mean 4.04, SD 0.64). The mean use of EMH before the COVID-19 pandemic was 38% (mean 0.38, SD 0.22), and it was 49% (mean 0.49, SD 0.25) during the pandemic. In total, 57% of the patient population was considered eligible for EMH. *Usefulness and benefits* (*β*=.440; *P*<.001), *Task perception* (*β*=.306; *P*=.001), and *Accessibility* (*β*=.140; *P*=.02) explained the intention to use EMH (*F*_3,131_=54.151; *P*<.001; *R*^2^=0.559). In turn, intention explained patient eligibility (*F*_1,130_=34.716; *P*<.001; *R*^2^=0.211), whereas intention and patient eligibility explained EMH use (*F*_2,129_=41.047; *P*<.001; *R*^2^=0.389). Patient eligibility partially mediated the relationship between intention to use EMH and EMH use, with a larger direct effect (*c*′=0.116; *P*<.001) than indirect effect (*c*=0.065, 95% CI 0.035-0.099; *P*<.001). Mental health counselors assessed patients’ eligibility for EMH mainly through the availability of computers and the internet and patient motivation.

**Conclusions:**

To stimulate the use of EMH, intention and patient eligibility need to be influenced. Intention, in turn, can be enhanced by addressing the perceived usefulness and benefits of EMH, perceived accessibility, and task perception. Access to a computer and patients’ motivation to use EMH are important in facilitating patient eligibility. To cause an impact with EMH in general practice, mental health counselors need to be convinced of the benefits of EMH and transfer this enthusiasm to the patient. It is recommended to involve mental health counselors in the development of EMH to increase the (perceived) added value and use.

## Introduction

### Background

The number of patients who visit their general practitioner (GP) with psychological problems is growing rapidly and, consequently, waiting lists for treatment are increasing [1]. For this reason, GPs and mental health care professionals search for ways to organize this care more efficiently. e–Mental health (EMH) care, often a combined approach with face-to-face care (ie, blended care), could be a solution [2]. This study applied the following definition for EMH: “The use of information and communication technologies for patients with mental health complaints or disorders to inform and/or support them in recovery from their mental health to ultimately improve quality of life” [3]. Interventions involve information and communication technologies, including treatment.

For the treatment of mental problems such as depression, there is convincing evidence of the effectiveness of EMH [4-7]. Furthermore, a growing number of patients are positive about the incorporation of new remote technologies in health care for their convenience and flexibility and the possibility of following treatment at their own pace [8]. In addition, the COVID-19 pandemic required reorganization of care as face-to-face contact was problematic and sometimes even impossible. To illustrate, 64% of Dutch GP practices started with videoconferences with patients during the COVID-19 pandemic [9]. However, the structural implementation of EMH is still limited and faces many difficulties [10-14].

### Mental Health Counselors and Adoption of EMH

In the Netherlands, mental health counselors (MHCs) working in general practices operate as gatekeepers in primary care concerning mental health problems [15,16]. MHCs have different educational backgrounds—approximately 50% are sociopsychiatric nurses, 20% are psychologists, and 15% are social workers [16]. These professionals treat patients with mild mental health problems and refer them to specialized care by licensed health care psychologists or psychotherapists in case of severe problems. They use EMH interventions as part of their tools to treat and coach patients. Although technical infrastructures and effective interventions are available, as well as sufficient reimbursement [17,18], actual use is low [10,11]. Facilitating future adoption and use requires insight into the most important underlying factors as well as the eligibility criteria MHCs use in their decision to apply EMH for their (vulnerable) patients. Hence, this study examined the use and nonuse of EMH by MHCs and aimed to unfold the underlying reasons and readiness to adopt EMH.

A study by Lokman et al [19] showed that 80% of GPs used EMH. Half of the GPs used EMH that was available via subscribed commercial eHealth platforms. The other half only referred patients to freely available self-help and psychoeducation websites. However, EMH was applied in <15% of the patients. According to the MHCs in that study, the currently available EMH was only suitable for one-third of their patients. Van der Vaart et al [15] revealed that MHCs applied EMH more often than psychologists in basic mental health care (49% vs 21%).

According to MHCs, important facilitators of EMH were the following: the perceived benefits, the perceived enhancement of tools it provides to coach and treat patients, the related enrichment for their own work, and its potential to improve the quality of care. In the long term, it can also save time as patients can proactively work through certain assignments and read or reread information at home [10]. Furthermore, MHCs considered themselves sufficiently digitally skilled and capable of providing EMH [11]. However, almost half of MHCs expressed the need for a decision aid and information on the effectiveness of EMH applications [11]. Impediments perceived by MHCs were as follows: the nonadherence of patients, the preference of patients for face-to-face contact, the insufficient possibilities MHCs perceive to be properly equipped to work with the eHealth platforms and the specific EMH applications, the mismatch between the supplied EMH materials and the patients’ needs, and the inflexibility of the EMH platform to attune the EMH content to the patients’ specific mental health problems and needs [11,20]. The most important reasons for the perceived mismatch were insufficient command of the Dutch language, low health literacy, and lack of a computer or low digital skills [10,11,20]. MHCs often related these reasons to a low level of education. Furthermore, experienced ambiguity in regulations for the reimbursement of EMH also negatively affected the behavior of MHCs, which was still the case in 2019 [21]. More studies have been conducted on facilitators of and barriers to EMH, although they were conducted among licensed psychologists [22] and psychotherapists and often concerned specialized long-lasting psychotherapy [23].

### Vulnerable Patients Visiting the MHC and Their Eligibility for EMH

Healthy life expectancy and the prevalence of chronic diseases and mental health problems are strongly socially patterned, disproportionately affecting individuals with a lower socioeconomic position [24]. These underprivileged individuals use EMH less frequently [25,26]. This is also the case for older adults [27-30] and individuals with severe mental health problems [31,32]. This is very undesirable and regrettable as EMH offers great opportunities given that interventions can be fluidly attuned to the needs of these specific groups through the presentation of bite-sized information in plain language accompanied by reading functions, appealing visuals and animations, and speech recognition [33]. As previously mentioned, and of utmost importance, the growing use of EMH may provoke further socioeconomic health inequality [25,34-36].

**Figure 1:**
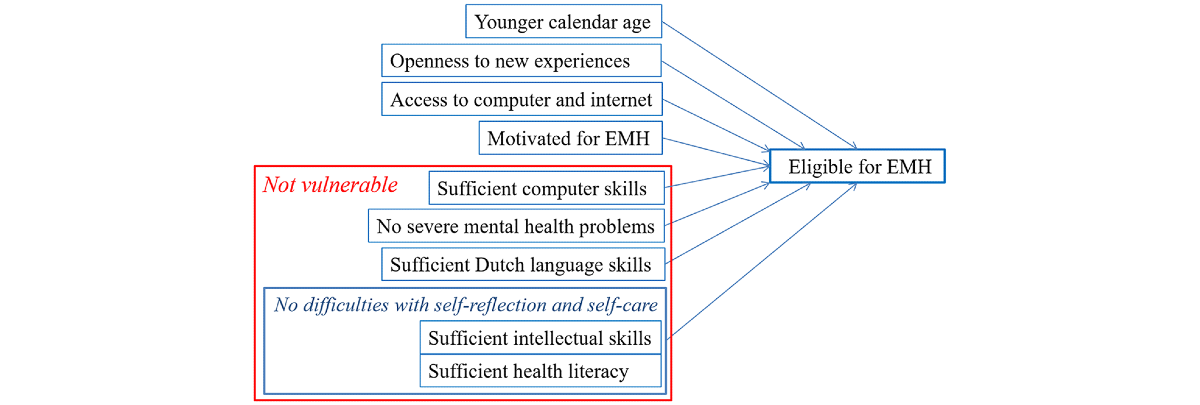
Eligibility criteria for the use of EMH. EMH: e–mental health.

In the literature, this low use of EMH by patients in lower socioeconomic positions and senior citizens is often associated with insufficient digital, health-related, and reading skills [20,25,37-39]. It is difficult for patients to find and use information via digital channels to adequately interpret and connect them with behavioral actions. As it is often an individual consideration and decision of the MHC whether EMH elements can be offered to a specific patient, it is important to gain insight into the criteria MHCs use in their consideration of patients’ eligibility for EMH. As there was no theoretical framework available at the time of the study, we developed one, as shown in Figure 1. We combined the aforementioned aspects of vulnerability from the studies by Krijgsman et al [10] and Wouters et al [11], items from the fit-for-blended care checklist [40], and relevant aspects derived from the studies by Titzler et al [12] and Osma et al [41] and from 3 orientational interviews with MHCs before our survey (unpublished).

### Theoretical Model of Factors Associated With Behavioral Intention and Use of EMH by MHCs

**Figure 2:**
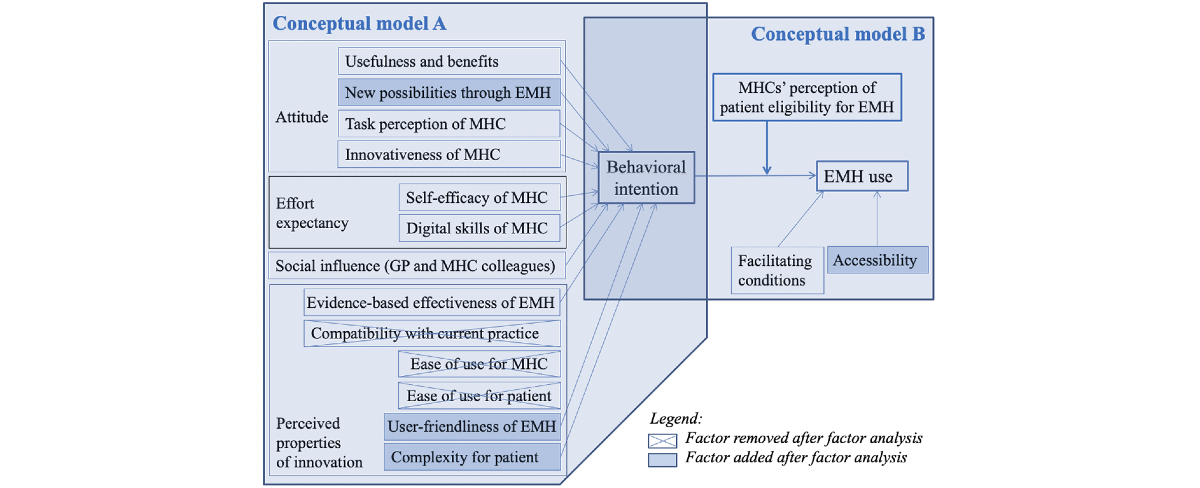
Theoretical model explaining the behavioral intention of the MHC to use EMH (conceptual model A) and MHCs’ actual use of EMH (conceptual model B). EMH: e–mental health; GP: general practitioner; MHC: mental health counselor.

To explain the behavioral intention and use of the MHC, a theoretical model was designed (Figure 2). The model was composed of elements from the reasoned action approach (RAA) model [42], the Unified Theory of Acceptance and Use of Technology (UTAUT) [43,44], the Measurement Instrument for Determinants of Innovation (MIDI) model [45], and the Diffusion of Innovation Theory [46]. In line with the RAA and UTAUT models, use of EMH is explained by behavioral intention to use EMH, whereas behavioral intention, in turn, is explained by the constructs *Attitude* (RAA), *Social Influence* (RAA and UTAUT) and *Self-efficacy* (RAA), *Effort Expectancy* (UTAUT), and *Perceived Usefulness* (UTAUT). With the aim of formulating a universal model, Venkatesh et al [43] integrated in the UTAUT elements from 8 models, including the RAA model, to explain the acceptance and use of IT in organizations. Although the UTAUT has been used extensively also to explain the introduction of eHealth [15,28,47,48], it has been criticized for being too restricted to describe the technology acceptance of individuals [30,49]. As we agreed with this criticism and wanted to develop a model that would show all the key factors influencing the considerations and decisions of MHCs, insights from diffusion and implementation theories were considered to be crucial additions. Hence, elements from the MIDI (*Characteristics of innovations*) [45] and Diffusion of Innovation Theory (*Compatibility with current practice*, *Relative advantage*, and *Complexity*) [46,50] were added, which led to further detailing of the rather general determinant *Attitude* and the selection of the factors of the construct *Perceived properties of innovations*. Figure 2 shows that our theoretical model, in line with the RAA and UTAUT, consists of 2 parts that will be empirically validated in this study. The first part explains the behavioral intention to use EMH (conceptual model A), and the second part explains the actual use of EMH by the MHC (conceptual model B).

Conceptual model B reflects that it will depend on the MHC’s assessment of the eligibility of the patient whether the behavioral intention is transformed into actual use of EMH. This could lead to a situation in which, although an MHC might be willing to use EMH, one could decide not to use it because of certain patient characteristics. This proposition is in line with the observation by Wouters et al [11] that MHCs are generally positive about EMH, although they apply it to a minority of their patients. On the basis of the UTAUT model [43,44] and previous studies [10,20,21,51], facilitating and impeding factors related to the organization of care in the GP practice were also considered as factors that directly influence the actual use of EMH. In line with Venkatesh et al [43], the construct *Facilitating conditions* was defined as the degree to which an individual believes that an organizational and technical infrastructure exists to support the use of the system. Examples of facilitating or impeding conditions were ambiguity in reimbursement and the availability of time, management support, and information on the innovation. Although our study used the same starting definition for *Facilitating conditions* as Venkatesh et al [43], the scale in the UTAUT questionnaire was operationalized in a different way. It measured not only the availability of resources and technical support (using the definition by Thompson et al [52]), as in the *Facilitating conditions* scale in our theoretical model (model B), but also aspects of the self-efficacy of the user [43].

### Focus of the Study

This study aimed to explain the behavioral intention and actual use of EMH by MHCs working in general practices. Most previous studies [10,11,18,19,53] described the use of EMH and the reasons for its use by MHCs, but they have not analyzed the correlations between barriers and facilitating factors on the one hand and behavioral intention and use on the other. Others did analyze the predictors of adoption readiness or behavioral intention [22,41,54] but did not assess EMH use itself. Our study addressed both the use of EMH and the factors that are associated with behavioral intention. Moreover, our study focused on MHCs and not (licensed) psychologists. To the best of the authors’ knowledge, no such studies have been conducted so far. An additional important aspect of our study is the specific interest in the application of EMH in vulnerable groups as the growing use of EMH might impede their access to health care to a greater extent compared with other groups [25,34-36].

### Hypotheses

This study tested 5 hypotheses.

The first hypothesis stated that MHCs with a high behavioral intention to use EMH score significantly higher compared with those with a low behavioral intention on the following 10 factors: (1) perceived usefulness and benefits of EMH, (2) task perception of the MHC, (3) innovativeness of the MHC, (4) social influence experienced by the MHC, (5) self-efficacy of the MHC toward the use of EMH, (6) digital skills of the MHC, (7) evidence-based effectiveness of EMH, (8) compatibility with current practice, (9) perceived ease of use of EMH for the MHC, and (10) perceived ease of use of EMH for the patient [15,22,41,43].

The second hypothesis proposed that perceived usefulness and benefits have the strongest association with intention to use EMH. The construct *Usefulness and benefits* is highly comparable with *Performance expectancy* of the UTAUT model. According to Venkatesh et al [43], this factor is proposed as the strongest predictor of behavioral intention to use technology in all technology acceptance models. Chismar and Wiley-Patton [55] confirmed the highest importance of perceived usefulness compared with social influence and ease of use in their empirical study.

The third hypothesis was that there is a significant correlation between the behavioral intention to use EMH and actual EMH use and that the strength of this relationship is largely determined by the assessment made by the MHC of the patients’ eligibility. Building on the findings that the intention to use EMH is much higher than actual EMH use and that patient characteristics might relate to this disparity, the third hypothesis asserts that the association between intention and use is moderated by the estimated patient eligibility for EMH.

Hypothesis 4 stated that facilitating and inhibiting organizational circumstances in the general practice also have a significant relationship with the use of EMH by the MHC [10,20,21,43,44,51].

According to hypothesis 5, the EMH eligibility assessment that MHCs conduct with regard to their patients includes primarily patient motivation to use EMH and the level of mental health problems. The hypothesized importance of patient motivation and the absence of disease-related contraindications (level of severity, lack of energy, lability, and suicidality) was based on the results of the qualitative study by Titzler et al [12]. Concerning the importance of severity, Osma et al [41] found therapists to have a positive intention to use EMH except in severe cases, such as psychosis, or if basic preconditions for the use of EMH are not met (eg, no internet access or insufficient literacy). Orientational interviews preliminary to our survey (unpublished) confirmed the importance of these factors.

As the validation of the questionnaire used to test the hypotheses was part of this study, the formulated hypotheses and developed model could undergo slight changes before the start of the analysis. The impact is discussed in the Methods section.

## Methods

### Research Design and Study Population

This was a cross-sectional study among MHCs using a web-based questionnaire (in LimeSurvey; LimeSurvey GmbH). Participants in the study had to be practicing MHCs working for at least 8 hours a week in a general practice.

### Recruitment Procedure

Convenience sampling was used. The Dutch eHealth platforms Ksyos, Therapieland, and Minddistrict sent a newsletter and email message to their customer field (total >1000) to invite potential participants. However, as this did not lead to a sufficient number of respondents even after sending a reminder, an advertisement on the web page of the National MHC Association and a LinkedIn message were added. To encourage participation, 5 vouchers worth €10 (US $10.32) were raffled.

### Ethical Considerations

The web-based survey was reviewed and approved by the Ethical Review Committee of the Open University before the start (U/2020/01469/MQF). All participants gave their informed consent before taking part.

### Questionnaire and Validation of the Questionnaire

#### Demographic Questions

The web-based questionnaire registered age, gender, type of general practice (with one or more MHCs), and educational background of the MHC (sociopsychiatric nurse, psychologist, social worker, or other) as well as the number of hours that the MHCs worked per week and their years of experience.

#### Factors Associated With Behavioral Intention and Use of EMH

In our model (Figure 2, model A), the behavioral intention to use EMH is the dependent variable that is explained by 10 independent variables. In this model (Figure 2, model B), behavioral intention to use EMH, in turn, is one of the 2 variables (together with the factor *Facilitating conditions*) explaining EMH use. To measure the behavioral intention to use EMH, we formulated 4 items in a similar way as for *Behavioral intention to use the system* in the UTAUT questionnaire [43]. The 4 items on this scale were scored by the MHCs on a 5-point Likert scale ranging from strongly disagree (1) to strongly agree (5). After item analysis, 3 of the 4 items remained, resulting in a reliable scale (3 items; Cronbach *α*=.85; Table 1).

In accordance with the theoretical model (conceptual model A; Figure 2), the 10 scales of the factors that are proposed to relate to behavioral intention to use EMH were measured as independent variables (*Usefulness and benefits*, *Task perception of the MHC*, *Innovativeness of the MHC*, *Social influence*, *Self-efficacy of MHC*, *Digital skills of the MHC*, *Evidence-based effectiveness of EMH*, *Compatibility with current practice*, and *Ease of use for the MHC and for the patient*). The factor *Facilitating conditions* was also measured as an independent variable, although it was directly related to EMH use (and part of conceptual model B; Figure 2).

To measure these 11 scales (ie, *Facilitating conditions* and the 10 scales of the factors that relate to behavioral intention), the items of the validated eMental Health Adoption Readiness scale by Feijt et al [56] were used as a starting point and complemented with items based on the MIDI questionnaire [45], the UTAUT questionnaire [43], a digital skill measurement tool, and own insights and literature (eg, related to the COVID-19 pandemic). To refine the items, our own scientific expertise [50,57,58] and input from practice were important. The latter input was collected from one of the authors working at Therapieland and by conducting 3 interviews with MHCs to collect information on important barriers, facilitators, and other considerations for the implementation of EMH and to check the content and completeness of our questionnaire. Before completion, the questionnaire was pretested among 8 MHCs. All items included in the scales were scored on a Likert scale. To validate the subscales of the questionnaire, these 11 scales were subjected to a principal factor analysis [59]. As the factor analysis led to some adjustments of the subscales (item composition, meaning, and sometimes factor name), the theoretical model and hypotheses were slightly modified. The changes in the theoretical model are shown in Figure 2. The factor analysis revealed that items of the scales *Usefulness and benefits of EMH* and *Compatibility with current practice* had to be recategorized in the revised scale of *Usefulness and benefits of EMH* and the new scale *New possibilities through EMH*. In addition, the items of the scales *Ease of use for the MHC* and *Ease of use for the patient* were redistributed, and the scales were renamed (*User-friendliness of EMH for both the MHC and the patient* and *Complexity for the patient*). After these adjustments, the measurement scales of the 10 factors that were assumed to influence behavioral intention showed a reasonable to good internal consistency (Cronbach *α*≥.68 and ≤.89), as shown in Table 1. After validation, the *Facilitating conditions* measurement scale was also adjusted. Its reliability was sufficient (5 items; Cronbach *α*=.78; Table 1). The content referred to organizational infrastructure with items on available time, finances, and administrative workload. In the factor analysis, *External obligation* and *Accessibility* emerged as new scales. The *External obligation* scale comprised 2 items that originally belonged to the *Facilitating conditions* scale. As this scale was not reliable (2 items; Cronbach *α*=.36; Table 1), it was not included in the revised hypothesized model (Figure 2). The *Accessibility* scale had good reliability (2 items; Cronbach *α*=.85; Table 1). *Accessibility* comprised 2 items of the original *Ease of use* scales and was interpreted as complementary to *Facilitating conditions* as it measured whether EMH was easy to obtain and use (because of technical infrastructure). It was presumed to have a direct relation to EMH use as its content came close to the definition of *Facilitating conditions* by Thompson et al [52]: “objective factors, ‘out there’ in the environment, that several judges or observers can agree make an act easy to do.”

For the factors from Table 1, mean scores were used in the regression model. The items that gave a negative evaluation of EMH were recoded. A high score (>3) indicates a positive evaluation by the MHC of that specific factor (eg, the MHC perceives EMH as useful and beneficial, and the MHC does not find that EMH uses complex language).

**Table 1:** Operationalization of the factors related to e–mental health (EMH) adoption readiness and use after factor analysis.

		Items, N	Cronbach *α*	Items
**Factors explaining intention to use EMH**				
	Usefulness and benefits of EMH	9	.89	EMH has advantages for the care I give. EMH does not improve the care I give. EMH has no added value for my work as MHC^a^. Using EMH treatment allows me to get faster results. Using EMH has added value for my patients. EMH is a nice addition to f2f^b^ contact. Patients come for f2f treatment, it takes a lot of effort to convince them of benefits of blended care. EMH fits well with how I am used to working. A patient who cooperates well in f2f therapy will generally also cooperate in EMH assignments.
	New possibilities of EMH	5	.75	Using EMH between f2f sessions makes the sessions more efficient. In the EMH programs, I can easily give feedback to the patients, and that has a motivating effect for the patient. EMH ensures that patients can read information about the treatment. Thanks to EMH, I can provide guidance to the patient even if the circumstances prevent me from making f2f appointments. If it is temporarily not possible to make f2f appointments, the treatment will continue through EMH.
	MHC task perception	3	.78	EMH is an indispensable part of the MHC work. EMH fits in well with my work as MHC. EMH does not fit the profession of MHC.
	Innovativeness of the MHC	5	.80	I am involved in setting up initiatives for the development of new EMH applications. Compared to colleagues, I often use EMH. Compared to colleagues, I take a lot of initiative in the field of EMH. I have ideas about what more could be developed in EMH applications (eg, virtual reality, gaming, biofeedback) In my work, I try to encourage colleagues to use EMH.
	Social influence	5	.73	My MHC colleagues use EMH. My GP^c^ uses the latest eHealth options. My GP expects me to use EMH when treating patients. My MHC colleagues expect me to use EMH when treating patients. Use of EMH is part of the policy of our general practice.
	Self-efficacy of the MHC	5	.81	Using EMH applications is easy for me. I still lack skills to give online therapy (via video calling). To start using EMH, I need to learn new skills. I need training in video calling. I need to practice to give empathetic written feedback.
	Digital skills of the MHC	4	.89	I can look up relevant information on the internet. I can work independently with a computer, and I am able to solve small problems myself. I can handle email messages well (receiving, sending, adding attachments). I can work well with digital documents: create, open, close, save in the correct folder, etc.
	Evidence-based effectiveness of EMH	2	.77	The EMH programs I work with have been proven to be effective. The EMH programs I work with are based on correct, scientific knowledge.
	User-friendliness of EMH	5	.69	The EMH programs are very user-friendly and invite the patients to use them. It is quite possible to use parts of the programs, the patient does not have to go through the entire program every time. I find the range of EMH programs clear. The EMH programs are very user-friendly and invite the MHC to use them. During the Covid pandemic, we are helped quickly and well by the suppliers of EMH.
	Complexity for patients	2	.68	Most EMH programs use difficult language. EMH is only suitable for people with a higher education.
**Factors explaining EMH use**				
	Facilitating conditions	5	.78	I do not get enough time from the GP to study the possibilities of EMH. The administrative burden prevents me from using EMH. It is impossible to declare the time it takes to delve into the possibilities of EMH. There is too little time in the f2f contacts to pay attention to EMH. Because the agenda is completely filled with f2f appointments, there is too little time to give good feedback to patients in the EMH platform.
	Accessibility	2	.85	I use EMH applications that are easily available. I use EMH applications that are easy to use.
	External obligation^d^	3	.36	I am obliged by the organization to use EMH. The Covid pandemic forces me to use EMH.
	Intention to use EMH	3	.85	I plan to use EMH as part of my treatments. I would like to supplement face-to-face contacts with EMH. After the Covid pandemic, I plan to continue using EMH.

^a^MHC: mental health counselor.

^b^f2f: face-to-face.

^c^GP: general practitioner.

^d^Not added to the theoretical model because of low reliability.

#### EMH Use and Patient Eligibility

EMH use and MHCs’ perception of patient eligibility were both operationalized as estimates by the MHC of the proportion of their patient population (1) who used EMH before the COVID-19 pandemic (ie, EMH use) and (2) whom they considered eligible for EMH (ie, patient eligibility). EMH use was measured using the question “Can you give an estimate of the proportion of patients for whom you have used eMH before the Covid pandemic?” MHCs’ perception of patient eligibility was measured using the question “Can you give an estimate of the proportion of your patients that are eligible for EMH?” Answering categories were 90% to 100%, 80% to 90%, 60% to 80%, 40% to 60%, 20% to 40%, 10% to 20%, and <10%. These population estimates were, in fact, alternative assessments as it was impossible in a regression model to relate the eligibility of an individual patient to the decision of the MHC to use EMH, as intended in the draft version of the conceptual model where EMH use was a dichotomous variable. To transform these categorical variables into ratio variables, the response categories were recoded as follows: <10% became 0.05, 10% to 20% became 0.15, 20% to 40% became 0.30, 40% to 60% became 0.50, 60% to 80% became 0.70, 80% to 90% became 0.85, and 90% to 100% became 0.95. The ratio variables were used in the regression model. Subsequently, the main reasons for (not) using EMH were asked using open questions. Respondents with increased use of EMH since the start of the COVID-19 pandemic were asked whether they expected that this increase would become permanent.

This study primarily aimed to explain EMH use before the COVID-19 pandemic. As the study started shortly after the beginning of the COVID-19 pandemic, some questions on the use of EMH during that period were also included. Social distancing in health care, as enforced by the Dutch government in March 2020, required GPs and MHCs to switch to remote patient contact as much as possible. This significantly increased the use of EMH in general practices [9].

### Analysis

All analyses were performed using SPSS software (version 26; IBM Corp). A 95% significance level was used for all tests (Cronbach *α*=.05) except for the 2-tailed *t* tests for hypothesis 1, where a significance criterion of *P*<.005 was used to correct for multiple testing (based on the Bonferroni correction).

To guarantee sufficient power for the regression analysis with 10 independent variables (m), the number of respondents (n), estimated by the rule of thumb of Tabachnick and Fidell [60], must exceed 130 (ie, 50+8 m).

The characteristics of the participants were analyzed using descriptive statistics. Correlation analyses were performed using all variables of the research model. To test hypothesis 1, a dummy variable was first calculated using a median split of EMH adoption readiness. Second, *t* tests were performed to reveal whether there were significant differences in the mean values of the 10 factors between respondents with high and low adoption readiness. To verify the second hypothesis, a multiple backward linear regression analysis was performed with behavioral intention as the dependent variable and the 10 factors from the research model (Figure 2, model A) as predictor variables.

The third hypothesis consisted of 2 parts. The analysis started with the second part: patient eligibility is a moderator variable in the association between behavioral intention and EMH use. This was investigated with a moderator analysis using the Hayes PROCESS module [61]—EMH use was the dependent variable (Y), behavioral intention was the independent variable (X), patient eligibility was the moderator variable (W), and *Accessibility* and *Facilitating conditions* were the covariates. Although this analysis also tested the first part of hypothesis 3 as well as hypothesis 4, a multiple linear regression analysis was also performed (backward) with EMH use as the dependent variable (Y) and behavioral intention, patient eligibility, and *Accessibility* and *Facilitating conditions* as independent variables as it facilitated the interpretation of the regression coefficients.

For the fifth hypothesis, descriptive statistics were used.

## Results

### Description of Participants

The web-based questionnaire was filled out 146 times between April 21, 2020, and August 8, 2020. A total of 4.8% (7/146) of the respondents did not give informed consent and, of the 139 respondents who did give informed consent, 7 (5%) were not active as MHCs. Hence, 132 questionnaires were available for the analysis.

Of the 132 respondents, 111 (84.1%) were female, and the mean age was 47.4 (SD 10.7) years. They worked between 8 and 40 hours per week as MHCs in one or more general practices (Table 2).

**Table 2:** Characteristics of the participants (N=132).

Characteristic		Values
Age (years), mean (SD; range)		47.4 (10.7; 27-65)
**Age (years; median s** **plit), median**		49.0
	Younger group (<50), n (%)	68 (51.5)
	Older group (≥50), n (%)	64 (48.5)
**Sex, n (%)**		
	Male	20 (15.2)
	Female	111 (84.1)
	Intersex	1 (0.8)
**Professional background, n (%)**		
	Psychologist	30 (22.7)
	Sociopsychiatric nurse	26 (19.7)
	Social worker	25 (18.9)
	Other^a^	51 (38.6)
Work hours per week, mean (SD; range)		23.0 (7.6; 8-40)
Number of patients per week, mean (SD; range)		25.6 (10.5; 4-65)
**Work experience (years), mean (SD; range)**		4.8 (3.5; 0-20)
	≤10, n (%)	128 (97)
	≤2, n (%)	45 (34.1)
**Number of MHC^b^ colleagues, n (%)**		
	None	42 (31.8)
**Platform used, n (%)**		
	Only Therapieland	41 (31.1)
	Only Minddistrict	80 (60.6)
	Therapieland and Minddistrict	9 (6.8)
	Neither Therapieland nor Minddistrict	2 (1.5)

^a^This group was very diverse and comprised, among others, psychiatric nurses, applied psychologists, orthopedagogists, and ergotherapeutists.

^b^MHC: mental health counselor.

### Variables and Correlation Analysis

In Multimedia Appendix 1, the variables of the research model—mean values and SDs—are listed as well as the Pearson correlation coefficients between them. All Pearson correlation coefficients were below the critical value of 0.80, confirming the independence of the constructs.

The correlation analysis showed that behavioral intention correlated significantly with 8 of the 10 scales. Behavioral intention had a strong positive correlation with *Usefulness and benefits of EMH* and *Task perception* and a medium correlation with *New possibilities*, *Innovativeness of the MHC*, *Social influence*, and *Evidence-based effectiveness*. The correlations between *User-friendliness* and *Complexity* were low.

### Hypothesis 1: Differences Between Groups With Low and High Behavioral Intention

Via a median split (median value of 4.0), the population was divided into a large group (98/132, 74.2%) with high behavioral intention (≥4; mean 4.32, SD 0.41) and a smaller group (34/132, 25.8%) with lower behavioral intention (<4; mean 3.25, SD 0.48). Using univariate *t* tests, the scores on the 10 factors were compared for the high– and low–behavioral-intention groups (Table 3). This partly confirmed the first hypothesis: MHCs with high behavioral intention scored significantly higher than MHCs with low behavioral intention on 6 of the 10 factors—*Usefulness and benefits of EMH*, *New possibilities through EMH*, *Task perception of the MHC*, *Innovativeness of the MHC*, *Social influence*, and *Evidence-based effectiveness of the EMH*—but not on perceived *Self-efficacy*, *Digital skills*, *User-friendliness*, or *Complexity* *of EMH*. These 6 factors were also the factors with a moderate to high correlation with EMH adoption readiness. The mean behavioral intention to use EMH in the total group was positive (mean 4.04, SD 0.64).

**Table 3:** Mean scale scores and differences for mental health counselors (MHCs) with low and high behavioral intention (BI).

	All MHCs, mean (SD)	MHCs with low BI, mean (SD)	MHCs with high BI, mean (SD)	*t* test (*df*)^a^	*P* value^b^
Usefulness and benefits of EMH^c^	3.80 (0.60)	3.19 (0.69)	4.01 (0.38)	−6.56 (40.2)^a^	<.001^a^
New possibilities through EMH	3.69 (0.60)	3.27 (0.53)	3.83 (0.56)	−5.12 (130)	<.001
Task perception of the MHC	4.02 (0.79)	3.29 (0.78)	4.27 (0.62)	−7.40 (130)	<.001
Innovativeness of the MHC	2.77 (0.76)	2.38 (0.82)	2.91 (0.69)	−3.67 (130)	<.001
Social influence	3.30 (0.66)	2.97 (0.63)	3.42 (0.63)	−3.55 (130)	.001
Evidence-based effectiveness of EMH	3.73 (0.58)	3.41 (0.72)	3.84 (0.49)	−3.19 (43.8)	.003^a^
Self-efficacy of the MHC	3.60 (0.73)	3.61 (0.77)	3.60 (0.72)	0.10 (130)	.93
Digital skills of the MHC	4.44 (0.59)	4.57 (0.51)	4.39 (0.61)	1.55 (130)	.12
User-friendliness for the MHC and the patient	3.58 (0.56)	3.48 (0.48)	3.61 (0.59)	−1.23 (130)	.22
Complexity for the patient	3.79 (0.65)	3.69 (0.69)	3.83 (0.64)	−1.04 (130)	.30

^a^Levene test for unequal variances; in all other cases, equal variances.

^b^*P*<.005 to control for multiple testing.

^c^EMH: e–mental health.

### Hypothesis 2: Factors Associated With Behavioral Intention to Use EMH

Factors associated with behavioral intention were examined using a multiple regression analysis (backward; Table 4). The first regression model (model 1; Table 4) had 10 independent variables, and these factors explained 56% of the behavioral intention (*F*_10,131_=15.113; *P*<.001; *R*^2^=0.555). However, only the scales on *Usefulness and benefits* (b=.401; *P*<.001) and *Task perception* (b=.339; *P*=.001) were significant. The last model (model 9; Table 4) explained 54% of the variance in behavioral intention (*F*_2,131_=76.102; *P*<.001; *R*^2^=0.541) with *Usefulness and benefits* (b=.471; *P*<.001) and *Task perception* (b=.316; *P*=.001). Hypothesis 2, which stated that the expected usefulness and benefits of EMH are the most important predictors of behavioral intention, was confirmed.

**Table 4:** Backward regression analysis to explain the behavioral intention to use e–mental health (EMH).^a^

			*R* ^2^		B (SE)		*β*		*P* value
**Model 1**			0.56						<.001
	Constant			0.852 (0.481)		N/A^b^		.08	
	Usefulness and benefits of EMH			0.425 (0.113)		.401		<.001	
	New possibilities through EMH			0.012 (0.080)		.011		.88	
	Task perception of the MHC^c^			0.274 (0.077)		.339		.001	
	Innovativeness of the MHC			−0.024 (0.065)		−0.028		.72	
	Social influence			0.034 (0.066)		.035		.61	
	Self-efficacy of the MHC			−0.057 (0.057)		−0.065		.32	
	Digital skills of the MHC			−0.012 (0.077)		−0.011		.88	
	Evidence-based effectiveness of EMH			0.054 (0.076)		.049		.48	
	User-friendliness for the MHC and the patient			0.089 (0.074)		.079		.23	
	Complexity for the patient			0.032 (0.065)		.033		.62	
**Model 9**			0.54						<.001
	Constant			1.120 (0.244)		N/A		<.001	
	Usefulness and benefits of EMH			0.499 (0.094)		.471		<.001	
	Task perception of the MHC			0.255		.316		.001	

^a^There was no multicollinearity (all variance inflation factor values were <4 and tolerance values were >0.20).

^b^N/A: not applicable.

^c^MHC: mental health counselor.

### Hypotheses 3 and 4: Factors Explaining EMH Use

EMH use before the COVID-19 pandemic (mean 0.38, SD 0.22) and patient eligibility (mean 0.57, SD 0.23) were determined as percentages of the patient population from the MHCs’ estimation.

Using the Hayes PROCESS module, a moderator analysis was performed with EMH use as the dependent variable (Y), behavioral intention as the independent variable (X), patient eligibility as the moderator variable (W), and *Accessibility* and *Facilitating conditions* as covariates. The model was significant (*F*_5, 126_=17.246; *P*<.001; *R*^2^=0.406), but the product term X×W was not (*F*_1, 126_=3.121; *P*=.08; *DR*^2^=0.015). Therefore, patient eligibility was not a moderator and, thus, the second part of the third hypothesis was rejected. However, the first part of the third hypothesis was confirmed—both *r*=0.55 (Multimedia Appendix 1) and the PROCESS regression analysis (b=.140; *P*<.001) showed a significant positive association between behavioral intention and EMH use. *Facilitating conditions* (mean 3.38, SD 0.77) and *Accessibility* (mean 3.77, SD 0.68) were not significantly related to EMH use, nor was the correlation significant (Multimedia Appendix 1). Hence, hypothesis 4 (*Facilitating conditions* and *Accessibility* have a significant relationship with EMH use) was rejected.

The backward multiple linear regression analysis that was also performed to facilitate the interpretation of the regression coefficients confirmed that only 2 variables were significant factors explaining EMH use: patient eligibility (b=.399; *P*<.001) and behavioral intention (b=.330; *P*<.001). Together, they explained 39% of the variance in EMH use (*F*_2,131_=41.047; *P*<.001; *R*^2^=0.389).

**Figure 3:**
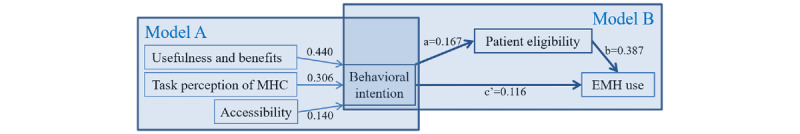
Empirically validated theoretical model with non-standardized regression coefficients. Model A shows the three significant factors of behavioral intention. Model B shows the results of the Hayes PROCESS mediation analysis of patient’ eligibility in the relationship between behavioral intention and EMH use (N=132). EMH: e–mental health; MHC: mental health counselor.

As patient eligibility was not a moderator variable but a direct scale of EMH use, the Hayes PROCESS module was used to examine whether it could be a mediator variable instead. The results are shown in Figure 3 (model B). Behavioral intention explained patient eligibility (*F*_1,130_=34.716; *P*<.001; *R^2^*=0.211), whereas behavioral intention and patient eligibility explained EMH use (*F*_2,129_=41.047; *P*<.001; *R*^2^=0.389). Hence, it was concluded that there was a partial mediation between behavioral intention and EMH use through patient eligibility. The direct effect (*c*’=0.116) of behavioral intention was larger than the indirect effect (*c*=0.065, 95% CI 0.035-0.099). All coefficients were significant, with *P*<.001.

### Verification of the Complete Model

To verify the complete theoretical model (ie, models A and B in Figure 2 combined), an additional multiple regression analysis (backward) was performed for behavioral intention. In addition to the *remaining* factors *Usefulness and benefits of EMH* and *Task perception of the MHC* (model 9; Table 4), the 2 factors for which a direct relationship with EMH use was assumed (*Facilitating conditions* and *Accessibility*) were included in the regression analysis. It turned out that the scales *Usefulness and benefits* (b=.440; *P*<.001), *Task perception* (b=.306; *P*=.001), and *Accessibility* (b=.140; *P*=.02) were significant explanatory factors of behavioral intention (*F*_3,131_=54.151; *P*<.001; *R*^2^=0.559). The empirically validated theoretical model in which only the significant factors related to EMH adoption readiness and use were considered is shown in Figure 3.

### Increased Use of EMH Since the Start of the COVID-19 Pandemic

The use of EMH before and since the start of the COVID-19 pandemic was compared. The mean use before the COVID-19 pandemic was 38.1% (mean 0.381, SD 0.219), and it increased to 49.4% after the start of the COVID-19 pandemic (mean 0.494, SD 0.247). There was a significant and strong correlation (*r*=0.794; *P*<.001) between use before and use since the start of the COVID-19 pandemic. More than half (70/132, 53%) of the MHCs had increased the use of EMH since the start of the COVID-19 pandemic, and this increase was also expected to be permanent for slightly more than half (36/70, 51%) of this group.

### Hypothesis 5: Factors Taken Into Account by MHCs in Assessing Patient Eligibility for EMH

**Figure 4:**
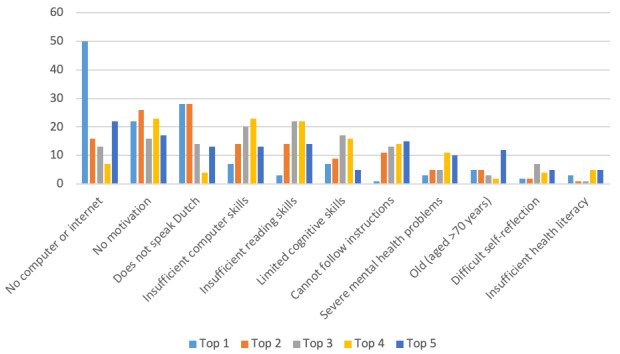
Number of times the characteristics were chosen as top-1 to top-5 that make a patient not eligible for e–mental health (EMH; n=131). Weighted total: no access to a computer- or the internet: 389, not motivated to work with EMH: 325, does not speak Dutch (and EMH programs are not available in their language): 315, insufficient computer skills: 210, insufficient reading skills: 195, limited cognitive skills: 159, cannot follow the instructions of the general practitioner or mental health counselor: 131, severe mental health problems: 82, old (aged > 70 years): 70, has difficulties with self-reflection: 52, and insufficient health literacy: 37.

The answers to the question asking to make a top 5 of the most important characteristics to be perceived as not eligible for EMH are summarized in Figure 4. In this graph, it is shown how often the items were chosen by respondents as top 1, top 2, top 3, top 4, and top 5. The order in which the characteristics are presented is considered the order of importance and is determined by the calculated weighted average score (weight factors: top 1×5, top 2×4, top 3×3, top 4×2, and top 5×1). A patient having no access to a computer or the internet was considered to be the most important reason to deem them not eligible for EMH. The total score for this characteristic was 389 (ie, 5×50+4×16+3×13+2×7+1×22, with 50 being the number of times it was chosen as top 1 by the respondents, 16 being the number of times it was chosen as top 2, and so on). *No motivation* came in the second position with a total score of 325, followed by *not speaking Dutch* (whereas EMH programs are offered in Dutch) with a total score of 315.

Regarding the question about the minimum properties or characteristics that a patient needs to have to be eligible for EMH, *access to a computer and the internet* was chosen 114 times, *motivation to work with EMH* was chosen 108 times, *openness to new experiences* was chosen 61 times, *reading and writing skills (in Dutch)* was chosen 57 times, *sufficient cognitive skills* was chosen 38 times, *discipline* was chosen 36 times, *demonstrable computer skills* was chosen 30 times, *other* was chosen 6 times, and *demonstrable health literacy* was chosen once.

The availability of a computer and the internet showed the highest scores in both assessments. When considering the patients’ attributes or skills, patient motivation for EMH use emerged as the most important factor. The severity of the mental health problems was given a low score. Therefore, the fifth hypothesis was partly rejected.

Many patient-related answers were given to the open questions about the main reason for (not) using EMH. This not only showed that the patient plays an important role in MHCs’ decision to use EMH, as was confirmed by the regression analysis, but also provided information about the factors that determine the assessment of the patients’ eligibility. Again, a lack of digital skills, limited language skills, and lack of motivation on the part of the patient were the predominant reasons. Interestingly, 10.6% (14/132) of MHCs mentioned the lack of a suitable EMH module for the patient in question.

## Discussion

### Principal Findings

This study aimed to unfold the underlying reasons of MHCs to adopt and use EMH as well as unveil the criteria MHCs use to estimate patient eligibility for EMH. The factor *Perceived usefulness and benefits* was the strongest predictor of behavioral intention to use EMH. In turn, behavioral intention had a direct and indirect effect (via estimated patient eligibility for EMH) on the use of EMH. To estimate patient eligibility for EMH, patients’ access to a computer and sufficient digital and language skills and motivation to use EMH were important.

In this study, the intention to use EMH was high, which is important to further disseminate and embed EMH use in practice as intention positively related to use in our study. The fact that intention to use EMH is high among MHCs is in line with previous findings in the Netherlands [10,11] but could be due to the broad definition of EMH and the fact that especially MHCs who are positive toward EMH might have been more likely to participate in this study as other studies have revealed that intentions varied between EMH applications [62] or were higher for the treatment of mild mental problems [17,63].

During the COVID-19 pandemic, EMH use was seen to increase from 38% on average to 49%; both percentages were already considerably higher than in 2016, when it was <15% [19]. More than half (70/132, 53%) of the MHCs reported increased use of EMH since the start of the COVID-19 pandemic, which was often expected to be permanent. It would be interesting to investigate over time whether this expectation also came true.

MHCs with a high behavioral intention scored significantly higher for 6 of the 10 factors (*Usefulness and benefits of EMH*, *New possibilities through EMH*, *Task perception of the MHC*, *Innovativeness of the MHC*, *Social influence*, and *Evidence-based effectiveness of EMH*) compared with those with a low EMH use intention. The results of the regression analysis stress the importance of *Usefulness and benefits of EMH*, *Accessibility*, and *Task perception* to increase MHCs’ intention to use EMH. *Usefulness and benefits of EMH* appeared to be the most important predictor of behavioral intention. *Accessibility* and *Task perception* were also predictive, although less strong. The primary importance of *Usefulness and benefits* is in line with relevant literature on the introduction of IT as well as in general [43] as applied to health care environments [15,55]. As a construct, *Usefulness and benefits of EMH* is closely related to *Performance expectancy* from the UTAUT model. *Performance expectancy* was defined as the extent to which a person believes that using the IT system will improve their work performance and is advantageous for patients, and it was also found to be the determinant variable of intention to use [43]. In applying the technology acceptance model, Chismar and Wiley-Patton [55] found that *Perceived usefulness* had a significantly strong effect on the intention of pediatricians to use internet-based applications, but *Subjective norm* and *Perceived ease of use* did not. Van der Vaart et al [15], who used the UTAUT model in their study on the use of web-based self-management interventions, also found *Performance expectancy* to be the most significant predictor of intention to use for both MHCs and primary care psychologists.

The second significant factor of behavioral intention in our study (ie, *Accessibility*) is not 100% equal to any of the scales of the UTAUT model, although it is related to *Effort expectancy*. *Effort expectancy* was defined by Venkatesh et al [43] as the degree of ease of use and is related to *Accessibility*, *User-friendliness*, and *Perceived self-efficacy* in our models A and B (Figure 2). Apparently, we need 3 factors in our model (Figure 2) to do justice to the different aspects of *Effort expectancy* by Venkatesh et al [43]. In the study by van der Vaart et al [15], *Effort expectancy* was a significant predictor of intention to use for MHCs but not for primary care psychologists. *Perceived ease of use* was not significant in the study by Chismar and Wiley-Patton [55] among pediatricians. These inconsistent results may be indicative of a construct that is formulated rather broadly. Still, there is clear consensus among the studies regarding the dominant role of perceived usability over ease of use. Only if users see added value in the use of EMH will they consider using it, and only then will ease of use become important [64].

The third significant factor of behavioral intention in our study (ie, *Task perception*) does not exist in the UTAUT model and, therefore, is not in the study by van der Vaart et al [15] either. However, *Task perception* relates very well to *Job relevance*, which was the factor determining perceived usefulness in the study by Chismar and Wiley-Patton [55]. In this study, the correlation between *Task perception* and *Usefulness and benefits* emerged as the strongest, confirming a close relationship.

It is interesting to note that *Social influence* was not a significant variable of behavioral intention, which is in line with the studies by van der Vaart et al [15] and Chismar and Wiley-Patton [55]. Seemingly, MHCs, primary care psychologists, and pediatricians make up their minds independent of others’ opinions and, therefore, do not need emphasis on influencing intentions to use EMH.

Correlation and regression analyses showed that MHCs with a higher behavioral intention were more likely to use EMH more frequently than their colleagues with a lower behavioral intention. The estimated eligibility of the patient population did not appear to be a moderator variable in the relationship between behavioral intention and EMH use. However, patient eligibility was found to be both a direct factor of EMH use and a mediator variable between behavioral intention and EMH use. The direct effect of patient eligibility on the use of EMH is larger than the direct effect of behavioral intention, which makes it the most determining factor in the decision of the MHC whether to use EMH. It is interesting, though unexpected, that the behavioral intention of MHCs influences their estimation of the eligibility of the patient population. This issue will be discussed later in this section. The question about the inverse relationship (ie, whether the patients’ eligibility influences behavioral intention) was not investigated as it is incompatible with the conceptual model. In this model, behavioral intention is a perception of the MHC that already exists before the MHC estimates the eligibility of the patient population involved.

The motivation of the patient emerged as the most important condition to consider a patient eligible for EMH in addition to access to a computer with internet and digital skills. Knowledge of the Dutch language and good reading skills were also high in ranking. The severity of the mental health problems was given a much lower ranking, which was unexpected considering the literature [12,41]. The fact that MHCs in our study considered the severity of the patients’ problems less of a barrier to using EMH may be explained by the fact that their average patient has mild to moderate mental problems compared with the patients with depression in the studies by Titzler et al [12] and Osma et al [41]. In the Netherlands, only mild to moderate mental problems are treated in general practice. Severe cases are treated in a specialized practice. The importance of motivation of the patient was confirmed in the answers to the open questions about the most important reasons for (not) using EMH in our survey and by the study by Wouters et al [11].

In this study, the MHCs estimated 57% of their patient population to be eligible for EMH. This is quite a high estimate compared with the 33% in the study by Lokman et al [19]. It is interesting that the intention of the MHCs to use EMH influences this estimate, as the mediation analysis revealed. This would mean that an MHC with a low intention to use EMH will be inclined to *disqualify* his patients for EMH. In this respect, the MHC can be considered an intermediary who plays a key role in the success of the implementation of EMH. From theory and experiments, it is known that success largely depends on the engagement of intermediaries or champions in the early stages of implementation [50,65,66]. Only an enthusiastic MHC will offer the intervention (EMH) to the patient and will be able to motivate them [12]. Owing to the emphasis that MHCs place on the motivation of the patient, the characteristics of vulnerable patients presumably received a lower ranking. Nevertheless, it became clear that MHCs will not yet use EMH for patients who lack computer and language skills.

### Study Limitations

Causal relationships were assumed in this study. However, a causal relationship cannot be established in this type of study. Nevertheless, as the research model is based on theoretical models that have already been empirically verified, this study does provide indications for the assumed causal relationships among EMH use, intention to use EMH, and their determinants.

A second limitation concerns the questionnaire, which, although based on validated questionnaires, was developed specifically for this study, which makes it difficult to compare results with the literature. On the positive side, the reliability of most of the scales proved to be good, and only small adjustments were needed to the predefined scales.

Third, the results of this study might be positively biased as convenience sampling was used and participants were mostly clients of Therapieland or Ksyos and Minddistrict. Obviously, MHCs who were positive about EMH and were already working on the web were more likely to complete the questionnaire than those who were less enthusiastic about web-based activities (*self-selection bias*). In our study, >98% (130/132, 98.5%) of the MHCs had access to a paid EMH platform, whereas, in the study by Lokman et al [19], approximately half of the GPs did not use purchased EMH. Unfortunately, the lack of insight into the response rate impeded a good estimate of the selection bias that might have occurred. A related issue is that the percentage of women in our sample was high (111/132, 84.1%). However, the occupation of MHC is female-dominated in the Netherlands. A recent (2021) Dutch publication revealed that 71% of MHCs are female [67].

Fourth, there may also have been response bias as self-report questionnaires with Likert scales were used that respondents filled in according to their own interpretation. A statement that is formulated in a fairly general way is perhaps all too easily answered with *agree*. This applies, for example, to the 2 items in the *Accessibility* scale. Feijt et al [56] came to the same conclusion for these items and removed them from the eMental Health Adoption Readiness scale for this reason. Although *Accessibility* is substantively related to *User-friendliness*, it emerged as a separate factor in the factor analysis. In spite of the fact that the reliability was good, there is some doubt regarding its content validity.

The final limitation concerns the difficult interpretation of the intention to use EMH, actual EMH use, and patient eligibility for EMH because of the broad definition of EMH we used (ie, ranging from means of communication to web-based treatment modules). Although the questions were formulated as specifically as possible by adding an example or by speaking of *EMH program* for therapy using web-based programs, the broad concept was used in the measurements of behavioral intention, EMH use, and patient eligibility. The research by van der Vaart et al [15] explicitly focuses on the use of a specific part of EMH (ie, the guided web-based self-management interventions in primary care). Therefore, the conclusions of that study are not entirely comparable with those described in this study.

### Recommendations for EMH Practice and Researchers

This study revealed that usefulness and benefits are by far the most important factors influencing the intention to use EMH. Only if potential users see added value in the use of an IT system will they consider using it, and only then will user-friendliness also become important [64]. Therefore, the EMH developer must ensure that EMH has added value for MHCs and patients. This is possible, for example, by developing EMH applications in cocreation with MHCs. Additional qualitative research would be ideally suited to obtain more specific input from MHCs (eg, to understand for which patients MHCs did not find a suitable EMH module, what modules are needed, and what characteristics they need to have). The research by Titzler et al [12] among psychotherapists is a good example of structured interviews that provide useful insights for EMH developers. It would also be good to actively involve the patients as that would help increase patient motivation while at the same time allowing for the determination of whether patient motivation is a real problem and not something that is wrongly estimated or negatively influenced by the MHC or whether the modules themselves are the problem. As the MHCs’ personal motivation to support patients with EMH influences their estimation of patients’ eligibility, it is important to make MHCs aware of this process and provide skill training on motivating patients regarding disease self-management and to use EMH. Motivational interviewing skills are posed as a helpful strategy.

This study also showed the importance of task perception of the MHC with regard to EMH. Government policy is necessary to structurally embed EMH in the task perception of the MHC (eg, by promoting digitalization in health care, providing the right reimbursement for EMH activities in general practice, and making EMH part of higher education curricula [68]).

The study results are also useful for the broader population of mental health care professionals, especially the findings with regard to eligible patients and the way in which the intention of health care professionals to use EMH affects their estimation of patient eligibility.

For future use of the questionnaire, specifically in questions on EMH use and patient eligibility, we recommend differentiating between the use of web-based communication and the use of web-based treatment modules. Better ways to determine the factors related to patient eligibility would also be worth investigating.

Recommendations for future research include a longitudinal study to verify the relationships we found in this study and investigate whether EMH use has maintained its upward trend after the COVID-19 pandemic. It would be especially interesting to further explore the influence of behavioral intention on perceived eligibility also among other professionals working in mental health care as it is important for practice to make professionals aware of the fact that personal motivation to support patients with EMH also influences the way in which patients’ motivation and skills to use EMH are estimated.

### Conclusions

It can be concluded that the intention to use EMH among MHCs was very positive. The most important factors explaining the intention to use were the perceived usefulness and benefits of the use of EMH followed by task perception and accessibility (ie, ease of getting started with it).

The relationship between behavioral intention to use EMH and actual EMH use was partially mediated by the perceived eligibility of the patient population. However, both the behavioral intention to use EMH and the estimated eligibility of the patient population had a significant and direct association with EMH use. The patients’ eligibility was most important, which means that an MHC will use little EMH if they consider the patient unsuitable for EMH even if the MHC is positive about the use of EMH.

To determine whether a patient is eligible for EMH, the patients’ access to a computer and the internet, digital skills, and Dutch language skills were primarily considered. In addition, patient motivation was found to be of utmost importance.

The study revealed that there will only be a future for blended care if the MHC is convinced of the added value of EMH and can transfer their enthusiasm to the patient.

## Appendix

Multimedia Appendix 1 Variables of the research model: mean, SD, and Pearson correlation coefficient.

## Abbreviations

EMH: e–mental health
GP: general practitioner
MHC: mental health counselor
MIDI: Measurement Instrument for Determinants of Innovation
RAA: reasoned action approach
UTAUT: Unified Theory of Acceptance and Use of Technology
