# Supplementary material for: Factors Associated With Intention and Use of e–Mental Health by Mental Health Counselors in General Practices: Web-Based Survey
Source: JMIR Form Res. 2022 Dec 20;6(12):e34754. doi: 10.2196/34754 (PMC9812270; doi:10.2196/34754)
Supplement: Multimedia Appendix 1 [file formative_v6i12e34754_app1.pdf]

Table S1. Variables of the research model: mean, SD, and Pearson correlation coefficient.

| Variable | Behavioral intention | Usefulness and benefits | New possibilities | Task perception | Inno-vativeness | Social influence | Self-efficacy | Digital skills | Ease of use | Complexity | Evidence -based effect | Facilitating conditions | Accessibility | EMH[a] use | Patient eligibility |
|---|---|---|---|---|---|---|---|---|---|---|---|---|---|---|---|
| **Measured on a 5-point scale (1-5)** | | | | | | | | | | | | | | | |
| Behavioral intention (mean 4.04, SD 0.64) | 1 | 0.70[b] | 0.43[b] | 0.66[b] | 0.41[b] | 0.32[b] | 0.02 | 0.03 | 0.26[b] | 0.20[b] | 0.37[b] | 0.17 | 0.33[b] | 0.51[b] | 0.46[b] |
| Usefulness and benefits (mean 3.80, SD 0.60) | N/A[c] | 1 | 0.56[b] | 0.74[b] | 0.54[b] | 0.39[b] | 0.06 | 0.01 | 0.28[b] | 0.24[b] | 0.44[b] | 0.32[b] | 0.28[b] | 0.51[b] | 0.61[b] |
| New possibilities (mean 3.69, SD 0.60) | N/A | N/A | 1 | 0.50[b] | 0.40[b] | 0.30[b] | 0.03 | −0.00 | 0.17[b] | 0.10 | 0.16 | 0.26[b] | 0.08 | 0.23[b] | 0.38[b] |
| Task perception (mean 4.02, SD 0.79) | N/A | N/A | N/A | 1 | 0.51[b] | 0.34[b] | 0.17 | 0.11 | 0.18[b] | 0.15 | 0.37[b] | 0.28[b] | 0.24[b] | 0.42[b] | 0.44[b] |
| Innovativeness (mean 2.77, SD 0.76) | N/A | N/A | N/A | N/A | 1 | 0.35[b] | 0.09 | 0.15 | 0.27[b] | 0.05 | 0.33[b] | 0.15 | 0.28[b] | 0.36[b] | 0.33[b] |
| Social influence (mean 3.30, SD 0.66) | N/A | N/A | N/A | N/A | N/A | 1 | 0.04 | −0.11 | 0.11 | 0.07 | 0.17 | 0.06 | 0.23[b] | 0.11 | 0.17 |
| Self-efficacy (mean 3.60, SD 0.73) | N/A | N/A | N/A | N/A | N/A | N/A | 1 | 0.35[b] | 0.11 | 0.08 | 0.03 | 0.28[b] | 0.07 | −0.06 | −0.02 |

| | | | | | | | | | | | | | | | | |
|---|---|---|---|---|---|---|---|---|---|---|---|---|---|---|---|---|
| | Digital skills (mean 4.44, SD 0.59) | N/A | N/A | N/A | N/A | N/A | N/A | N/A | 1 | 0.17 | 0.29[b] | 0.10 | −0.01 | 0.12 | 0.02 | 0.04 |
| | Ease of use (mean 3.58, SD 0.56) | N/A | N/A | N/A | N/A | N/A | N/A | N/A | N/A | 1 | 0.21[b] | 0.23[b] | 0.24[b] | 0.26[b] | 0.23[b] | 0.27[b] |
| | Complexity (mean 3.79, SD 0.65) | N/A | N/A | N/A | N/A | N/A | N/A | N/A | N/A | N/A | 1 | 0.16 | 0.23[b] | 0.07 | 0.16 | 0.42[b] |
| | Evidence-based effect (mean 3.73, SD 0.58) | N/A | N/A | N/A | N/A | N/A | N/A | N/A | N/A | N/A | N/A | 1 | 0.08 | 0.30[b] | 0.22[b] | 0.36[b] |
| | Facilitating conditions (mean 3.38, SD 0.77) | N/A | N/A | N/A | N/A | N/A | N/A | N/A | N/A | N/A | N/A | N/A | 1 | −0.08 | 0.09 | 0.19[b] |
| | Accessibility (mean 3.77, SD 0.68) | N/A | N/A | N/A | N/A | N/A | N/A | N/A | N/A | N/A | N/A | N/A | N/A | 1 | 0.15 | 0.17 |
| **Measured as a percentage of the population** | | | | | | | | | | | | | | | | |
| | EMH use (mean 38%, SD 22%) | N/A | N/A | N/A | N/A | N/A | N/A | N/A | N/A | N/A | N/A | N/A | N/A | N/A | 1 | 0.55[b] |
| | Patient eligibility (mean 57%, SD 23%) | N/A | N/A | N/A | N/A | N/A | N/A | N/A | N/A | N/A | N/A | N/A | N/A | N/A | N/A | 1 |

[a]EMH: e–mental health.

[b]*P*<.05 (2-sided testing).

[c]N/A: not applicable.
